# Synthesis and Biological Evaluation of a γ-Cyclodextrin-based Formulation of the Anticancer Agent 5,6,11,12,17,18,23,24-Octahydrocyclododeca[1,2-*b*:4,5-*b’*:7,8-*b’’*:10,11-*b’’’*]tetraindole (CTet)

**DOI:** 10.3390/molecules15064085

**Published:** 2010-06-04

**Authors:** Simone Lucarini, Mauro De Santi, Francesca Antonietti, Giorgio Brandi, Giuseppe Diamantini, Alessandra Fraternale, Maria Filomena Paoletti, Andrea Tontini, Mauro Magnani, Andrea Duranti

**Affiliations:** 1 Dipartimento di Scienze del Farmaco e della Salute, Università degli Studi di Urbino “Carlo Bo” I-61029 Urbino, Piazza del Rinascimento 6, Italy; E-Mails: simone.lucarini@uniurb.it (S.L.); francesca.antonietti@uniurb.it (F.A.); giuseppe.diamantini@uniurb.it (G.D.); andrea.tontini@uniurb.it (A.T.); 2 Dipartimento di Scienze Biomolecolari, Università degli Studi di Urbino “Carlo Bo” I-61029 Urbino, Via Aurelio Saffi 2, Italy; E-Mails: mauro.desanti@uniurb.it (M.D.S.); giorgio.brandi@uniurb.it (G.B.); alessandra.fraternale@uniurb.it (A.F.); maria.paoletti@uniurb.it (M.F.P.); mauro.magnani@uniurb.it (M.M.)

**Keywords:** indole-3-carbinol, indole cyclic tetramer, γ-cyclodextrin, breast cancer

## Abstract

5,6,11,12,17,18,23,24-octahydrocyclododeca[1,2-b:4,5-b’:7,8-b’’:10,11-b’’’]tetraindole (**CTet**), an indole-3-carbinol (**I3C**) metabolite endowed with anticancer properties, is poorly soluble in the solvents most frequently used in biological tests. This study indicates that the use of γ-cyclodextrin (γ-CD) avoids this problem. Formulated with γ-CD **CTet** is a potent inhibitor of DNA synthesis in both estrogen receptor positive (MCF-7) and estrogen receptor negative (MDA-MB-231) human breast cell lines (IC_50_ = 1.20 ± 0.04 μM and 1.0 ± 0.1 μM, respectively).

## 1. Introduction

**Figure 1:**
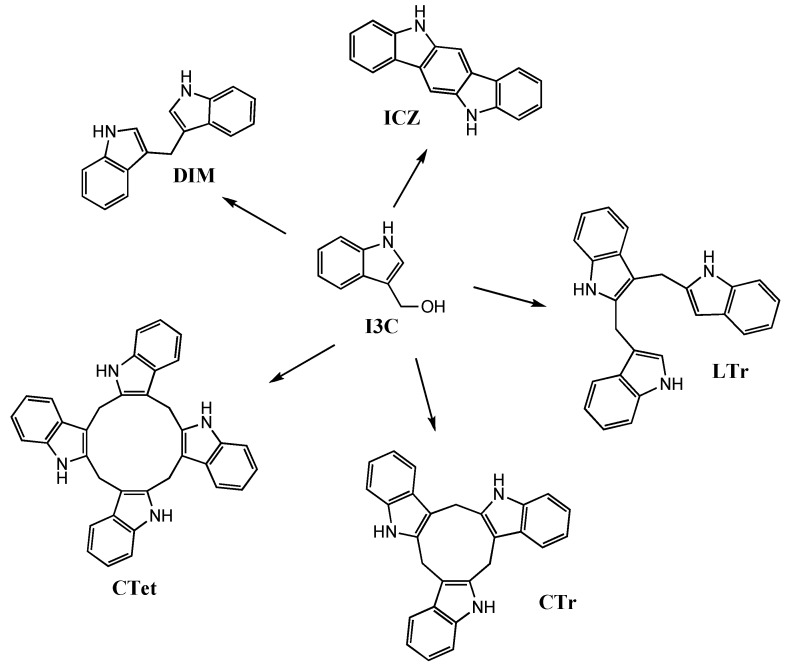
Chemical structures of compounds **I3C**, **DIM**, **ICZ**, **LTr**, **CTr**, and **CTet**.

Edible cruciferous vegetables of the genus *Brassica* are endowed with chemopreventive and chemotherapeutic properties [1,2,3]. These actions depend on an autolysis product of 3-indolylmethyl glucosinolate (glucobrassicin), namely indole-3-carbinol (**I3C**, Figure 1) [4,5], and the resulting indole oligomers produced in the acidic environment of the stomach: 3,3’-diindolylmethane (**DIM**) [4,5], indolo[3,2-*b*]carbazole (**ICZ**) [6,7], the linear trimer **LTr** [6,8,9], the cyclic trimer **CTr** [6,8,10], and the cyclic tetramer **CTet** [11,12] (Figure 1) [2]. The current interest of pharmacologists and medicinal chemists in this topic has resulted in several reports which disclosed synthetic analogues of **I3C** [13,14,15], **DIM** [16,17,18,19,20,21,22,23,24,25,26,27,28,29,30,31], and **CTr** [32] possessing anticancer properties.

In order to study the antitumor effects of **CTet**, we needed a reliable and gram-scale synthesis of this compound. The methods reported in the literature for the preparation of **CTet** are three. The first involves the one-pot reaction of indole and formaldehyde in acidic methanol, and is in principle advantageous [33]. The second is in fact aimed at obtaining **CTr** and affords **CTet** as a by-product in low yield after recrystallization with DMSO; this method, employing gramine as a source in a basic environment of the presumed 3-methylene indolenine intermediate, is practical and efficient but not all its reagents could be utilized on a large scale synthesis because of their toxicity [34]. The third protocol utilizes **I3C** and acetic acid but the acidic conditions [8] and the purification by means of silica gel column chromatography lead to the formation of polymers and degradation products, so it is not possible to isolate pure **CTet** by recrystallization with methanol [12].

Unfortunately, **CTet** is poorly, if at all, soluble in the most common solvents, in particular those usually employed in biological experiments (acetone: 0.04, pyridine: 0.22, 2-butanol: 0.11, DMSO: 0.1% w/v). Furthermore, in chloroform, ethanol, methanol, and toluene **CTet** solubility is less than 0.1% w/v and the compound is insoluble in water and physiological saline solutions. Several procedures were therefore evaluated to increase **CTet** solubility in a pharmaceutically acceptable formulation. We found the approach with γ-cyclodextrin (γ-CD) promising, therefore it was selected for further investigation.

## 2. Results and Discussion

**Scheme 1:**
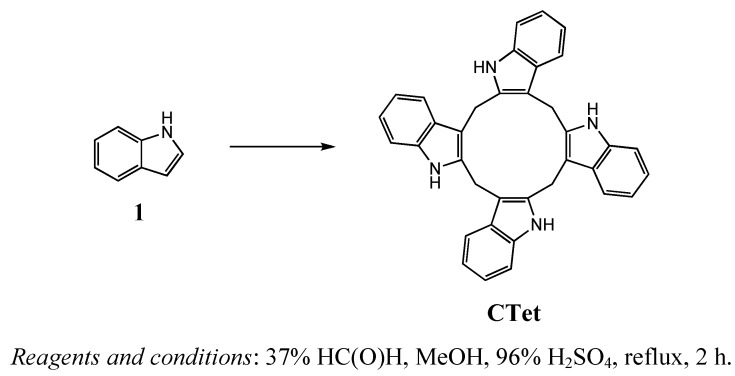
Synthesis of 5,6,11,12,17,18,23,24-octahydrocyclododeca[1,2-*b*:4,5-*b’*:7,8-*b’’*:10,11-*b’’’*]tetraindole (**CTet**).

The synthesis of pure **CTet** was carried out by modifying Bergman *et al*.’s procedure [33]. When we applied this protocol, we repeatedly obtained results not congruent with the reported ones. In particular, the precipitate that separated from the mixture contained only a trace of the desired **CTet**, being instead constituted of numerous side-products, probably formed through polymerization processes. However, the filtrate of the reaction mixture did contain **CTet**, which was isolated by chromatography and recrystallization. In addition, HPLC analysis of the chromatographic fractions showing a single spot on TLC plates demonstrated that **CTet** was present together with **CTr**. Bergman’s protocol was modified by prolonging reaction time, due to the presence of the starting material in the mixture after one hour, and by purifying the crude by two rapid passages through short aluminum oxide columns. **CTet** was finally obtained with a purity higher than 99% by recrystallization from acetone, rather than pyridine [33] and DMSO [34], to facilitate solvent removal. The protocol proved to be scalable, in that it was possible to run it using up to 150 mmol of indole (17.5 g); these experiments gave yields and **CTr**/**CTet** ratios comparable with those reported on a lower scale (amounts of reagents higher than those reported were not used) (Scheme 1).

**Figure 2:**
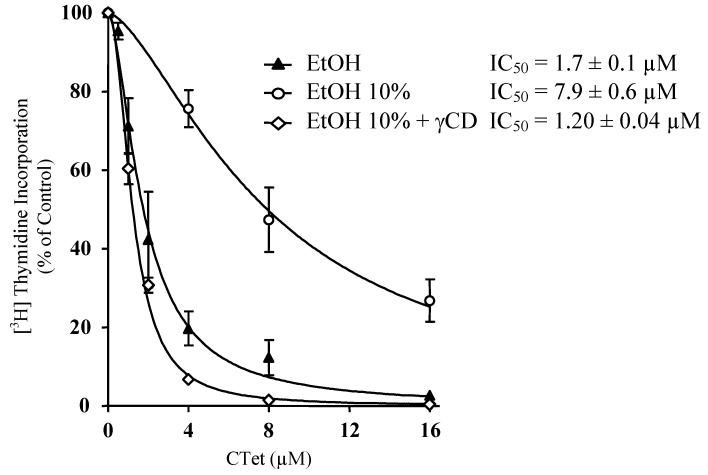
Effect of **CTet** formulated in aqueous solutions on DNA synthesis of MCF-7 breast cancer cell line. Cells were treated with various concentrations of **CTet** suspended in 10% EtOH (Ο), 10% EtOH with 160 mM γ-CD (◊) or pure EtOH (▲); during the last 5 h of treatment, cells were pulsed with [^3^H]thymidine, and the incorporation into DNA was determined (1.5 μCi). Data are expressed as percentage of cells treated with vehicle only and are means ± SEM of at least three experiments.

With the aim of examining the antiproliferative activity of **CTet**, the drug was solubilized in pyridine or suspended in ethanol or DMSO and tested on estrogen receptor positive (ER+) breast cancer cell line MCF-7. It resulted that **CTet** in pyridine could not affect cell proliferation, whereas **CTet** in DMSO did in a dose-dependent manner (IC_50_ = 11.3 ± 1.4 µM). Also, **CTet** suspended in ethanol showed good antiproliferative activity in the same cell line (IC_50_ = 1.7 ± 0.1 μM) (Figure 2). A pure ethanolic preparation, however, could not be used in clinical studies, thus we considered important to investigate formulations of **CTet** in an aqueous system.

Several protocols such as Solvent Induced Activation (SIA) system with PVP-Cl (polyvinylpyrrolidone-Cl) in different mediums, HP-55 (hydroxypropyl methyl cellulose phthalate), and β- or γ-CD complexation, were investigated. Only γ-CD formulation gave encouraging results. So, while the suspension obtained by diluting the **CTet** mixture in ethanol/water 1:10 showed a significant loss of biological activity (IC_50_ = 7.9 ± 0.6 μM; *P* < 0.001) (Figure 2), when dilution of **CTet** was carried out in a γ-CD EtOH/H_2_O (1:10) solution, the activity of **CTet** resulted superimposable to that of **CTet** suspended in pure ethanol (IC_50_ = 1.20 ± 0.04 µM) (Figure 2).

**Figure 3:**
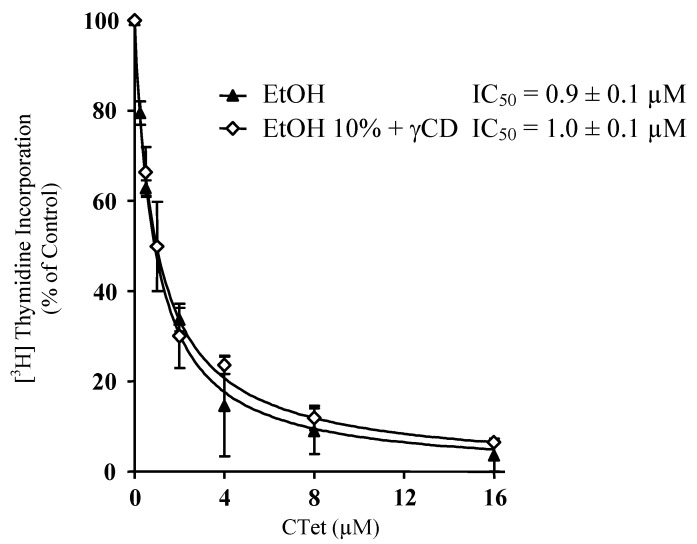
Effect of **CTet** formulated in aqueous solutions on DNA synthesis of MDA-MB-231 breast cancer cell line. Cells were treated with various concentrations of **CTet** suspended in 10% EtOH with 160 mM γ-CD (◊) or pure EtOH (▲); during the last 5 h of treatment cells were pulsed with [^3^H]thymidine, and the incorporation into DNA was determined (1.5 μCi). Data are expressed as percentage of cells treated with vehicle only and are means ± SEM of at least three experiments. A 10% ethanolic solution of γ-CD did not have any appreciable cytotoxicity in our tests.

The antiproliferative activities of **CTet** both suspended in pure ethanol and formulated in γ-CD 10% ethanol were also tested on an estrogen receptor negative (ER-) breast cancer cell line (MDA-MB-231); the results were comparable with those obtained with MCF-7 cells (IC_50_ = 0.9 ± 0.1 and 1.0 ± 0.1 µM, respectively) (Figure 3). Notably, a 10% ethanolic solution of γ-CD did not have any appreciable cytotoxicity in our tests.

Finally, we had ascertained by HPLC that these formulations were stable for many months at room temperature in the dark; this observation is corroborated by the fact that antiproliferative tests in MCF-7 cells were comparable with those reported above (data not shown).

## 3. Experimental

### 3.1. General

All reagents were purchased from Sigma-Aldrich or Carlo Erba with the exception of PVP-Cl and HP-55 which were furnished by Eurand, β-cyclodextrin (CAPTISOL^®^, CyDex), and γ-cyclodextrin (CAVAMAX^®^ W8, Wacker); they were in the highest quality commercially available. Solvents were RP grade. Melting points were determined on a Büchi B-540 capillary melting point apparatus. The structure of **CTet** was unambiguously assessed by MS, ^1^H-NMR, and ^13^C-NMR. MS (ESI) spectra were recorded with a Waters Micromass ZQ spectrometer in a positive mode using a nebulizing nitrogen gas at 400 L/min and a temperature of 250 ºC, cone flow 40 mL/min, capillary 3.5 Kvolts and cone voltage 60 V; only molecular ion in positive ion mode [M+H]^+^ is given. Retention time (*t*_R_) value was determined by direct HPLC analysis by Waters 2795 Separations Module, Alliance HT and Waters 2996, Photodiode Array Detector spectrometers with a Supelcosil^TM^ LC-18 (15 cm × 4 mm, 3 μM; Supelco) column using a combination of acetonitrile and aqueous solution 0.1% formic acid as eluent. ^1^H-NMR and ^13^C-NMR spectra were recorded on a Bruker AC 200 or 50, instrument, respectively, and analyzed using the WIN-NMR software package. Chemical shifts were measured by using the central peak of the solvent. Purification of the crude material was carried out by column chromatography on aluminum oxide (0.05–0.15 mm, Fluka). TLC analyses were performed on precoated aluminum oxide on aluminum sheets (60 F_254_, neutral; Merck).

### 3.2. Synthesis of 5,6,11,12,17,18,23,24-octahydrocyclododeca[1,2-*b*:4,5-*b’*:7,8-*b’’*:10,11-*b’’’*]tetraindole *(**CTet**)*

To a solution of indole (3.12 g, 26.7 mmol) and aqueous 37% HC(O)H (3.2 mL, 40 mmol) in CH_3_OH (240 mL), 96% H_2_SO_4_ (1.74 mL) was added. The mixture was stirred at reflux in the dark for 1 h, then further HC(O)H (3.2 mL, 40 mmol) was added, the mixture was stirred in the same conditions for 1 h, cooled to room temperature and concentrated in the dark. Purification of the solid by two short, protected from light, and fast aluminum oxide column chromatographies (cyclohexane/EtOAc 6:4, *R_f_* = 0.82) and washing with hot CH_3_OH gave a white solid consisting (HPLC/MS) in a 9:1 mixture of **CTr** and **CTet** [HPLC: Supelcosil^TM^ LC-18; flow: 0.5 mL/min; λ_max_: 284 nm; eluent: CH_3_CN/aqueous solution 0.1% HCOOH with a gradient 7:3 to 9:1 in 9 min; *t*_R_ **CTr**: 4.95 min, *t*_R_ **CTr**: 6.93 min]. Yield: 31% (1.08 g). Recrystallization [(CH_3_)_2_CO, 78 mL] afforded pure **CTet** as a white solid. Mp: chars over 300 ºC. MS (ESI) *m/z*: 517.2 [M+H]^+^. ^1^H-NMR [(CD_3_)_ 2_CO]: δ3.88 (s, 8H, CH_2_), 6.85 (dd, 4H, ArH, *J_1_* = 7.0 and *J_2_* = 8.0 Hz), 6.99 (dd, 4H, ArH, *J_1_* = 7.0 and *J_2_* = 8.0 Hz), 7.24 (d, 4H, ArH, *J* = 8.0 Hz), 7.33 (d, 4H, ArH, *J* = 8.0 Hz), 9.95 (s, 4H, NH); ^13^C-NMR (pyridine-*d*_5_): δ 23.6, 109.1, 112.6, 119.4, 120.4, 122.0, 131.4, 137.7, 138.0.

### 3.3. CTet formulations

A suspension of **CTet** (0.0083 g, 0.016 mmol) in pure EtOH (1 mL) was magnetically stirred at room temperature for different times (1 to 3 days, 1,000 rpm). The highest percentage of inhibition was obtained when the suspension was stirred for at least 2 days. This time was routinely used in all further experiments. The emulsion obtained was then diluted (volume ratio 1:10) by an aqueous solution of γ-CD (177 mM); the resulting white emulsion had a final concentration of 1.6 mM. The antiproliferative assays were performed with 10 μL of formulated product appropriately diluted in 1 mL of the cellular culture medium.

### 3.4. Cell cultures and antiproliferative assay

The human breast carcinoma ER+ (MCF-7) and ER- (MDA-MB-231) cell lines were cultured in DMEM (Dulbecco’s Modified Eagle’s Medium) supplemented with 10% FCS (Fetal Calf Serum), 2 mM L-glutamine, 10 g/L NEAA (Non-Essential Amino Acid), 50 mg/L streptomycin, 1,000 U/L penicillin, with (in the case of MCF-7) or without (in the case of MDA-MB-231) 10 mg/L insulin. Cells (30,000/well in 24-well tissue culture plates) were treated with the several **CTet** formulations or respective vehicles for 72 h, and during the last 4 h of treatment were pulsed with 1.5 μCi of [^3^H]thymidine and processed [12].

### 3.5. Statistical analyses

Data are means ± SEM of at least three separate experiments. Differences between means were evaluated by Student *t*-test; differences were considered significant at *P* < 0.05 (Prism5, GraphPad Software Inc., La Jolla, CA, USA).

## 4. Conclusions

A straightforward, reproducible, and scalable synthesis of **CTet** is reported, together with a formulation of **CTet** that allows the molecule to exert its pharmacological potential as an inhibitor of DNA synthesis in both ER+ and ER- human breast cancer cells. It is hypothesized that γ-CD is capable to enhance the otherwise very low solubility of the drug in aqueous systems.
